# Complete necrosis of graft ureter following renal transplant in a patient with primary antiphospholipid syndrome: A case report

**DOI:** 10.1002/ccr3.1620

**Published:** 2018-05-29

**Authors:** Manoj Hilary Fernando, Umesh Jayarajah, Donald Rubakan, Ruwan Fonseka, Serozsha Goonewardena

**Affiliations:** ^1^ Department of Urology National Hospital of Sri Lanka Colombo Sri Lanka; ^2^ Department of Vascular and Transplant Surgery National Hospital of Sri Lanka Colombo Sri Lanka

**Keywords:** allograft pelvis‐ native ureter ureteropyeloplasty, antiphospholipid syndrome, case report, necrosis of graft ureter

## Abstract

Complete necrosis of the graft ureter is a rare but serious complication following kidney transplant. In a patient with antiphospholipid syndrome, a combination of factors such as arterial thromboembolism, hematoma formation, and surgical collateral damage can cause ischemia of the graft ureter. Preoperative optimization of disease activity and coagulation with meticulous preservation of ureteric perfusion may help in prevention.

## 1 INTRODUCTION

Urological complications following renal transplantation are known to occur in 2.5%‐30% of all graft recipients.^1^ However, complete necrosis of the graft ureter is rare and only few cases have been reported.^2^, ^3^ Early necrosis of the graft ureter is a serious complication which can cause allograft failure or infected collections resulting in considerable morbidity and mortality.^3^ Patients with antiphospholipid syndrome are particularly at a higher risk because of the increased tendency for vascular thromboembolism, due to the disease and hemorrhage, due to anticoagulation.

We describe a 38‐year‐old male with primary antiphospholipid syndrome who underwent renal transplant which was complicated by complete necrosis of the graft ureter which was successfully repaired.

## 2 CASE PRESENTATION

**Figure 1:**
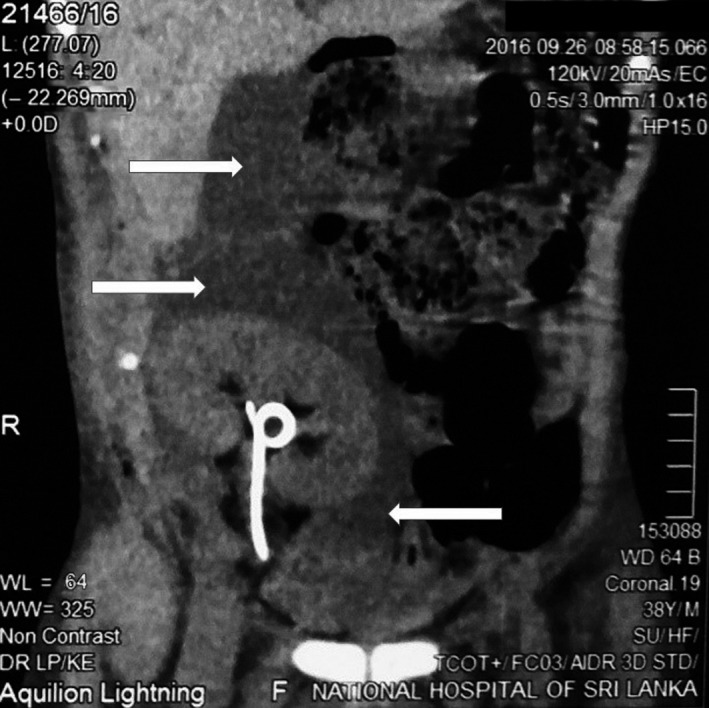
Noncontrast Computed tomography scan showing homogenous fluid collection (white arrows) posterior to the transplanted kidney which extends superiorly up to the right subhepatic area and inferiorly into the pelvis, displacing the bladder to the left

A 38‐year‐old male underwent a tissue‐matched renal allograft transplant for end‐stage renal failure secondary to hypertension and primary antiphospholipid syndrome. Primary antiphospholipid syndrome was diagnosed 2 years prior to renal transplantation when he developed recurrent episodes of thrombosis of the arteriovenous fistula and neck veins, for which he was started on warfarin. Anticoagulation was optimized prior to surgery by withholding warfarin 5 days before surgery while bridging with heparin. During the early postoperative period (first postoperative week), he developed a peri‐renal hematoma (ultrasonography 13 cm × 7 cm). Noncontrast computed tomography scan detected a homogenous fluid collection posterior to the transplanted kidney which extended superiorly up to the right subhepatic area and inferiorly into the pelvis, displacing the bladder to the left (Figure 1). Anticoagulation was then withheld and he recovered over the next few days with normalization of serum creatinine and urine output. Prior to discharge, his urethral catheter and ureteric stent were removed and warfarin was restarted.

Three weeks after the surgery, he presented with reduced urine output and progressive abdominal distension associated with pain and fever. Ultrasound scan revealed a large perigraft collection. Resuscitation and urgent exploration revealed a viable graft with a large urinoma posterior to the kidney. A possible anastomotic leak was suspected and a passive external drain was placed to allow adequate drainage of urine and healing of the anastomotic site. However, the conservative approach failed and there was no reduction in the drain output. Therefore, surgical exploration and definitive reconstruction were planned.

**Figure 2:**
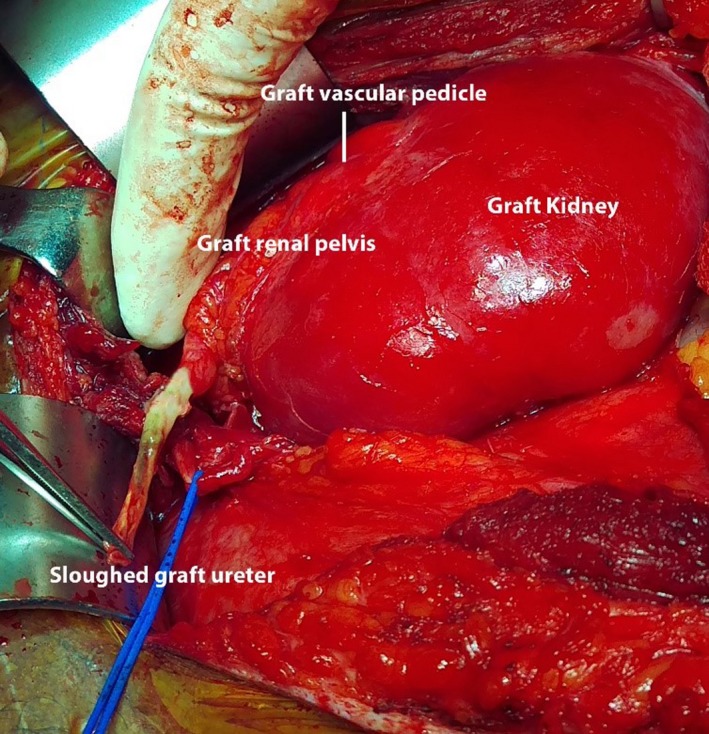
Figure showing the sloughed off graft ureter

**Figure 3:**
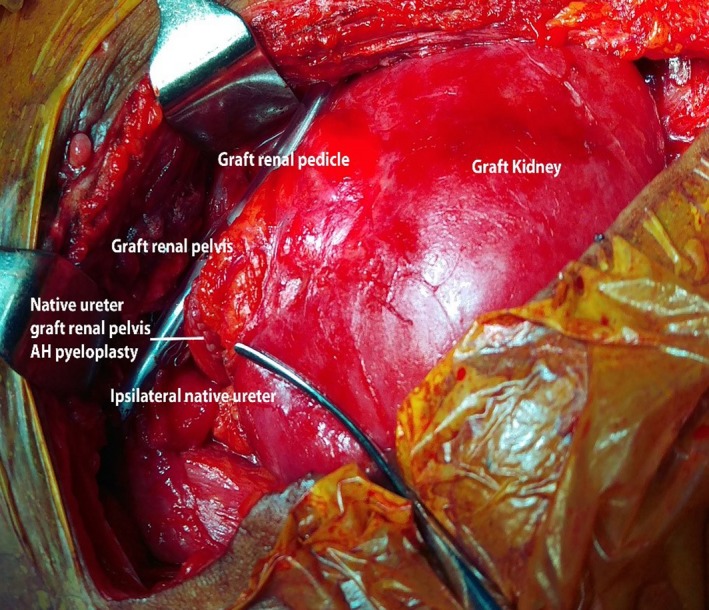
Figure showing the native ureter‐graft renal pelvis Anderson‐Hynes pyeloplasty

**Figure 4:**
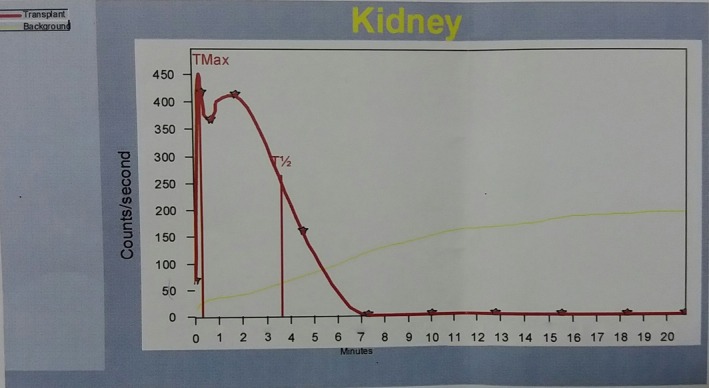
Figure showing the excretion phase of the 99 m‐Technetium DTPA renogram

Surgical exploration revealed an allograft vascular pedicle in the inferolateral aspect of the graft with a completely sloughed off allograft ureter (Figure 2). Sloughed part of the allograft ureter was excised up to the pelviureteric junction where the graft renal pelvis appeared well‐perfused and healthy. Free flow of urine from the graft was noted. Ipsilateral native ureter was divided close to the native renal pelvis and approximated to the graft extraperitoneally, posterior to the vas deferens. Allograft pelvis‐ native ureter ureteropyelostomy was performed over a 6 French double J stent connecting the native ureter and the allograft renal pelvis, essentially similar to the technique described for the Anderson and Hynes dismembered pyeloplasty (Figure 3). Recovery following surgery was uneventful with no evidence of obstruction or allograft failure. Six months following surgery, he developed a perirenal abscess around the graft kidney, which was successfully drained. A 99 m‐Technetium DTPA (diethylene‐triamine‐pentaacetate) renography was carried out 9 months after surgery, which showed normal uptake and excretion with no evidence of obstruction (Figure 4). At 1 year following the reconstructive surgery, the patient had good urine output with a stable serum creatinine at 1.2 mg/dL.

## 3 DISCUSSION

Complications of the urinary tract following kidney transplantation is known to cause considerable morbidity and mortality.^4^ The commonest urinary tract complication in renal transplants is ureteral stricture with a reported incidence of 1%‐10%.^3^ Partial ureteric necrosis and leakage of urine were reported to occur in 1%‐5% of renal transplants.^3^ Fortunately, complete necrosis of the graft ureter is encountered rarely.

Antiphospholipid syndrome is an autoimmune disorder in which autoantibodies (antiphospholipid antibodies) including lupus anticoagulant and anticardiolipin antibodies, are directed against phospholipid‐binding proteins. It is clinically characterized by an increased tendency for recurrent vascular thromboembolism involving arteries and veins.^5^, ^6^ This generalized hypercoagulable state can potentially result in thrombosis, and may affect any segment of the vascular bed or solid organ, such as the liver, spleen, pancreas, kidney, and intestine. Furthermore, it may cause thrombotic microangiopathy, due to microvascular endothelial injury, intimal expansion and fibrin deposition resulting in microvascular thrombosis.^5^, ^6^, ^7^ Moreover, thrombotic complications are higher in end‐stage renal disease patients with antiphospholipid syndrome.^7^ Thus, to prevent such thrombotic complications, anticoagulation therapy is recommended for patients with antiphospholipid syndrome.^5^, ^7^ However, studies have shown that allograft thrombosis can develop despite anticoagulation.^5^, ^6^, ^7^ Furthermore, anticoagulation increases the risk of bleeding which can also lead to graft loss. Our patient developed a serious but rare complication which was complete necrosis of the graft ureter. The reason for this is probably multifactorial. The early postoperative hematoma due to anticoagulation therapy and the traction imposed by the displacement of the bladder to the contralateral side may have compromised the blood supply of the graft ureter due to direct pressure on to the vessels. Moreover, the hypercoagulable state due to antiphospholipid syndrome may have resulted in increased susceptibility for thrombosis in the small blood vessels supplying the graft ureter, particularly when it was compressed by a surrounding hematoma. Furthermore, overzealous dissection and surgical handling of the graft ureter may have resulted in damage to small blood vessels increasing the susceptibility for thrombosis and worsening of ischemia.

Management of defects in the graft ureter depends on the extent and severity. Smaller defects such as a simple anastomotic leak may require primary suture of the leaking segment over a double J stent insertion or a reimplantation of the ureter. However, complete necrosis due to ischemia requires total replacement of the graft ureter and in such cases, exploration and definitive treatment should not be delayed.^8^, ^9^ Several surgical techniques have been described to restore the continuity of the urinary tract. One such technique is the ureteropyelostomy or pyelo‐pyelostomy using the native ureter. The technique was first described by Carpus et al and then by Wagner et al^10^, ^11^ The use of the contralateral native ureter has been described by Mahdavi and Gholamrezaie.^11^

The utilization of the physiologic anti‐reflux mechanism of the bladder is a known advantage in this technique. Furthermore, it is relatively easy to perform and if carefully dissected, the blood supply of the native ureter could be preserved. This procedure requires a normal native ureter. If the native ureter is nonfunctional or absent due to previous surgeries or abnormally short, other alternative techniques such as direct vesicopyelostomy may be performed.^12^, ^13^, ^14^

However, in rare instances, difficulty in mobilizing the bladder or the graft kidney could make the procedure technically difficult. In such cases, a Boari flap is a useful alternative.^15^

However, vesicopyelostomy may result in considerable vesico‐ureteric reflux. Therefore, reflux of urine into the graft renal pelvis may occur resulting in complications such as graft failure or recurrent infections. These infections are difficult to control due to the immunocompromised state of the host. Furthermore, it is shown that reflux is less common in patients with Boari flap compared with direct vesicopyelostomy.^14^ In cases of longer ureteric defects, more complex surgical procedure may be required. Ileal interposition and vesico‐psoas hitch and Boari flaps are utilized in such instances.^14^, ^16^, ^17^

Our patient with antiphospholipid syndrome developed a hematoma in the early postoperative period, which was managed conservatively. Later, he developed a complete necrosis of the graft ureter and a large urinoma. Multiple factors may have contributed to the compromised blood supply of the graft ureter, which is solely derived from the graft renal artery. Careful attention to perioperative optimization, meticulous haemostasis and preservation of peri‐ureteric and pelvi‐ureteric adipose tissue with the contained vasculature may help to prevent this complication.

## 4 CONCLUSION

Ischemic sloughing of the entire ureter following renal transplant is fortunately a rare occurrence and is generally attributed to the compromised ureteric blood supply. In a patient with antiphospholipid syndrome, complete necrosis could result due to a combination of factors such as arterial thromboembolism, hemorrhage and surgical collateral damage leading to small vessel occlusion and ischemia. Preservation of graft ureteric blood supply with meticulous haemostasis may help to prevent this complication. Furthermore, following an early postoperative hematoma, possibility of a subsequent necrosis of the graft ureter should be considered and close monitoring and follow up becomes mandatory.

## CONFLICT OF INTEREST

None declared.

## AUTHOR CONTRIBUTION

DMHF, UJ, and BADR: contributed to the collection of information and writing of the manuscript. WRDF and SASG: contributed to writing and final approval of the manuscript.

## CONSENT

Informed written consent was obtained from the patient for publication.
